# 619 Utilization of a Designated Burn Case Manager Reduces Length of Stay After Burn Injury

**DOI:** 10.1093/jbcr/iraf019.248

**Published:** 2025-04-01

**Authors:** Taylor Ross, Herbert Phelan, Victoria Miles, Jeffrey Carter, Jonathan Schoen

**Affiliations:** Burn Center at University Medical Center; Louisiana State University Burn Center; University Medical Center New Orleans Burn Center; Louisiana State University Burn Center; Louisiana State University Health Sciences Center - New Orleans

## Abstract

**Introduction:**

Burn patients’ discharge planning is more complex than most, so much so that it should be thought of as a perishable skill. Due to staffing shortages, our burn unit’s discharge planning needs were met by a rotating pool of Licensed Clinical Social Workers (LCSWs) who would cover the burn service for periods of time ranging from several hours to several weeks before rotating off to other parts of the hospital. After advocating for the position we were granted a designated burn case manager (DBCM), an LCSW whose primary responsibility would be to the burn unit. We undertook a quality improvement project to assess the impact of a DBCM on length of stay (LOS) after burn injury.

**Methods:**

A DBCM was designated for our burn unit beginning 01 Oct 2022. Prior to that date, the DBCM in question had no experience with burn injury, treatment regimens, or burn discharge planning. We queried our burn registry performance improvement database for all patients admitted with an acute burn injury with or without concomitant trauma who spent at least 1 midnight in the hospital between 01 Oct 2020 to 30 Sep 2022 (PRE-DBCM) and 01 Oct 2022 to 30 June 2024 (POST-DBCM). Patients were excluded if they died during the index admission, signed out against medical advice, or were admitted with an exfoliative skin disorder or necrotizing soft tissue infection. Patients for whom hospice was arranged as a disposition prior to their death were allowed to remain in the analysis. LOS was normalized as days per percent total body surface area (TBSA) burned. As the data was skewed, all values are reported as medians with interquartile ranges and analyzed with Mann-Whitney-U tests. Alpha was set at 0.05. Daily hospital costs for this time period were taken from our institution’s financial administrative data.

**Results:**

Four hundred thirty subjects were in the overall cohort (PRE-DBCM n=210, POST-DBCM n=220). Age and TBSA between the cohorts were similar (p=ns). Median LOS was 1.46 [0.85, 2.67] days/%TBSA burned in the PRE-DBCM cohort and 1.23 [0.67, 2.43] days/%TBSA burned in the POST-DBCM cohort (p=0.049). Given that a day in our hospital cost $3,200 during the time period of this QI project, utilization of a DBCM resulted in a savings to the hospital of $1.89 million USD.

**Conclusions:**

The burn-specific knowledge and resources required to provide optimal burn case management are most easily acquired when the job is performed on a daily basis. Designating one case manager to be responsible to the burn unit results in a significantly shorter LOS and a financial savings to the institution that more than offsets the cost of the hire.

**Applicability of Research to Practice:**

Due to the financial solvency, the hiring of a dedicated case manager is something that every unit should consider.

**Funding for the Study:**

N/A

